# Different subregional metabolism patterns in patients with cerebellar ataxia by ^18^F-fluorodeoxyglucose positron emission tomography

**DOI:** 10.1371/journal.pone.0173275

**Published:** 2017-03-20

**Authors:** Minyoung Oh, Jae Seung Kim, Jungsu S. Oh, Chong Sik Lee, Sun Ju Chung

**Affiliations:** 1 Department of Nuclear Medicine, Asan Medical Center, University of Ulsan College of Medicine, Seoul, Korea; 2 Department of Neurology, Asan Medical Center, University of Ulsan College of Medicine, Seoul, Korea; Kyungpook National University School of Medicine, REPUBLIC OF KOREA

## Abstract

We evaluated cerebellar subregional metabolic alterations in patients with cerebellar ataxia, a representative disease involving the spinocerebellum. We retrospectively analyzed ^18^F-fluorodeoxyglucose positron emission tomography (^18^F-FDG PET) images in 44 patients with multiple system atrophy of the cerebellar type (MSA-C), 9 patients with spinocerebellar ataxia (SCA) type 2, and 14 patients with SCA type 6 and compared with 15 patients with crossed cerebellar diaschisis (CCD) and 89 normal controls. Cerebellar subregional metabolism was assessed using 13 cerebellar subregions (bilateral anterior lobes [ANT], superior/mid/inferior posterior lobes [SUPP/MIDP/INFP], dentate nucleus [DN], anterior vermis [ANTV], and superior/inferior posterior vermis [SUPV/INFV]) to determine FDG uptake ratios. MSA-C and SCA type 2 showed severely decreased metabolic ratios in all cerebellar subregions compared to normal controls (ANT, 0.58 ± 0.08 and 0.50 ± 0.06 vs. 0.82 ± 0.07, respectively, *p* < 0.001). SCA type 6 showed lower metabolic ratios in almost all cerebellar subregions (ANT, 0.57 ± 0.06, *p* < 0.001) except INFV. Anterior-posterior lobe ratio measurements revealed that SCA type 2 **(**Right, 0.81 ± 0.05 vs. 0.88 ± 0.04, *p* < 0.001; Left, 0.83 ± 0.05 vs. 0.88 ± 0.04, *p* = 0.003) and SCA type 6 (Right, 0.72 ± 0.05 vs. 0.88 ± 0.04, *p <* 0.001; Left, 0.72 ± 0.05 vs. 0.88 ± 0.04, *p* < 0.001) showed preferential hypometabolism in the anterior lobe compared to normal controls, which was not observed in CCD and MSA-C. Asymmetric indices were higher in CCD and MSA-C than in normal controls (*p* < 0.001), whereas such differences were not found in SCA types 2 and 6. In summary, quantitative analysis of cerebellar subregional metabolism ratios revealed preferential involvement of the anterior lobe, corresponding to the spinocerebellum, in patients with cerebellar ataxia, whereas patients with CCD and MSA-C exhibited more asymmetric hypometabolism in the posterior lobe.

## Introduction

Cerebellar ataxia is clinically defined as a class of neurodegenerative disorders characterized by progressive degeneration of cerebellum and is often accompanied by a variety of neurological and systemic symptoms. Achieving a correct diagnosis in ataxic patients remains a challenge due to the wide phenotypical overlap among various hereditary and non-hereditary ataxias. Spinocerebellar ataxia (SCA), which is composed of several subtypes, constitutes the main group in the genetic classification of autosomal dominant cerebellar ataxia [1]. However, hereditary ataxias can also manifest as sporadic disorders with adult onset [2]. For instance, cases with later onset of a pure cerebellar syndrome due to a mutation in *SCA6* may lack an obvious family history [1]. Multiple system atrophy of the cerebellar type (MSA-C) is the most common cause (30%) of sporadic late-onset cerebellar ataxia [3]. However, clinical diagnosis of MSA is not highly accurate [4, 5]. For instance, Kim *et al*. reported that the ratio of patients with SCA mutations was high among those that met the diagnostic criteria for MSA based on clinical features, especially cerebellar dysfunctions [6]. Furthermore, up to 20% of patients with sporadic adult-onset ataxia harbor a causative gene mutation, including a *de novo* mutation, despite a negative family history. Nevertheless, due to their heterogeneity, accurate diagnosis of cerebellar ataxias remains a clinical challenge [7, 8].

Pathologically, several SCA and MSA-C subtypes share the comparable feature of loss of neurons, including Purkinje cells. Recent advances in genetic analysis, however, revealed that the distribution of neuronal damage in cerebellar cortex and deep nuclei differs among SCA subtypes [9], which is distinct from characteristic cytoplasmic inclusion bodies in neuronal cytoplasm found in MSA-C [10]. A growing body of evidence indicates that different cerebellar subregions are likely to be affected even during the preclinical stages of various spinocerebellar ataxia subtypes [11]. From these reports, we hypothesized that subregional metabolism in cerebellum were different among patients with distinct cerebellar ataxia pathologies.

While cerebellar metabolic alterations caused by SCA and MSA-C were previously demonstrated using ^18^F-fluorodeoxyglucose positron emission tomography (^18^F-FDG PET) [12–14], specific subregional metabolic patterns of these disorders have not yet been established. Several technical obstacles remain in the investigation of the complex structure of cerebellum; however, recent advances in scanner resolution facilitate the identification of various cerebellar structures based on the orientation of cerebellar fissures used as important landmarks [15]. Furthermore, we recently proposed an improved spatial normalization method using a cerebellum-specific template for the quantification of glucose metabolism by PET [16].

Several approaches for segmentation of the cerebellum have been proposed to accurately characterize cerebellar anatomy and function [17, 18]. Among functional subsystems, damage to the spinocerebellum, which anatomically corresponds to anterior vermis and paravermis of the anterior cerebellar lobe, results in characteristic cerebellar ataxia [19]. Thus, we analyzed relative cerebellar subregional metabolism rates with a focus on the spinocerebellum in patients with cerebellar ataxia and compared with those obtained from patients with crossed cerebellar diaschisis (CCD) and normal controls to assess potential differences in subregional cerebellar metabolism rates specific to different cerebellar diseases.

## Materials and methods

### Subjects

We retrospectively reviewed 44 patients with MSA-C, 9 patients with SCA type 2, and 14 patients with SCA type 6 who underwent ^18^F-FDG PET imaging at our institution. This cohort included all patients with SCA diagnosed between March 2006 and September 2013. Among those with SCA, 9 patients with SCA type 2 and 3 patients with SCA type 6 were included in our previous work [16]. Patients with MSA were diagnosed based on the consensus statement [20], were classified as probable MSA-C by movement disorder specialists (S.J.C. and C.S.L.), underwent ^18^F-FDG PET imaging between March 2006 and December 2010, and were followed for more than two years. All SCA patients were molecularly confirmed to harbor CAG trinucleotide repeat expansions of corresponding *SCA* genes. Age, sex, age at onset, duration from onset to ^18^F-FDG PET imaging, clinical symptoms, unified Parkinson’s disease (PD) rating scale (UPDRS) scores (for MSA-C patients), and number of CAG repeats (for SCA patients) were obtained from the medical records, if available (Table 1).

**Table 1:** Demographic features of the study groups.

	Normal (n = 89)	CCD (n = 15)	MSA-C (n = 44)	SCA type 2 (n = 9)	SCA type 6 (n = 14)
**Sex (M:F)**	55:34	7:8	24:20	4:5	8:6
**Age (y)**	49.9 ± 14.0	49.3 ± 11.3	55.9 ± 7.0	38.2 ± 12.7 ^a^	58.3 ± 9.4 ^b^
**Age of onset (y)**		56.2 ± 6.9	52.3 ± 10.8	31.4 ± 12.8 ^a^	54.2 ± 7.4
**Time from onset to PET (y)**			1.9 ± 1.8	6.9 ± 4.2 ^b^	4.2 ± 4.2
**Time from onset to last F/U (y)**			4.7 ± 2.0	7.7 ± 4.3	5.1 ± 4.5
**UPDRS III scores**			14.5 ± 11.0		
**Urinary incontinence (n)**			15	0	2
**Orthostatic hypotension (n)**			19	1	2
**Ataxia (n)**			40	9	12
**Tremor (n)**			5	4	4
**Pyramidal symptom (n)**			9	1	1
**Dysarthria (n)**			33	9	11
**Number of CAG repeats (mean ± SD)**				42.1 ± 3.3	22.9 ± 1.5

^a^ MSA-C vs. SCA type 2, *p <* 0.001

^b^ SCA type 2 vs. SCA type 6, *p =* 0.001

Fifteen patients with CCD due to brain tumors in the frontal cortex who were identified by ^18^F-FDG PET imaging were also included as controls for reduced cerebellar metabolism by non-ataxic causes. Additionally, 89 nine healthy controls between the ages of 17 and 85 years were selected from the normal ^18^F-FDG PET data pool maintained at our institution. Healthy controls were confirmed to have no neurological or psychological diseases. This study was approved by the institutional review board of Asan Medical Center (IRB No.2014-0023) with the need for informed consent waived and is in accordance with the declaration of Helsinki on ethical principles for medical research involving human subjects.

### ^18^F-FDG PET image acquisition

All ^18^F-FDG PET scans were acquired using an ECAT HR+ scanner (CTI-Siemens, Knoxville, Tennessee, USA) in 159 subjects and using a Discovery 690 scanner (GE healthcare, Waukesha, Wisconsin, USA) in 12 subjects. All subjects fasted for a minimum of six hours before ^18^F-FDG PET imaging.

In patients studied by the ECAT HR+ scanner, transmission scans of 5 minutes using a rotating pin source of germanium-68 for attenuation correction and emission scans of 15 minutes were acquired 40 minutes after intravenous injection of 370 MBq ^18^F-FDG. Patients lay under resting conditions with eyes closed in a room with dim light. In-plane and axial resolutions of the scanner were 4.3 mm and 8.3 mm full width at half maximum (FWHM), respectively. Following acquisition, ^18^F-FDG PET images were reconstructed using a Gaussian filter (FWHM, 2 mm) and displayed in a 128 × 128 matrix (pixel size, 1.72 × 1.72 mm with a slice thickness of 2.43 mm). Emission images were reconstructed with ordered subset expectation maximization (OSEM) using 16 subsets and 6 iterations.

In patients studied by the Discovery 690 scanner, computed tomography data for attenuation correction were acquired first, followed by three-dimensional ^18^F-FDG PET image acquisition. In-plane and axial resolutions of the scanner were 4.9 mm and 5.6 mm FWHM, respectively. ^18^F-FDG PET images were reconstructed using a Gaussian filter (FWHM, 2 mm) and displayed in a 256 × 256 matrix (pixel size, 0.98 × 0.98 mm with a slice thickness of 3.27 mm). Emission images were reconstructed with OSEM using 24 subsets and 2 iterations.

### ^18^F-FDG PET image analysis

We analyzed ^18^F-FDG PET images using volumes of interests (VOIs) and voxelwise comparisons.

### Preprocessing and spatial normalization

^18^F-FDG PET images were spatially normalized using Statistical Parametric Mapping 2 (SPM2, Wellcome Department of Cognitive Neurology, Institute of Neurology, University College London, United Kingdom) in conjunction with MATLAB version 2013a (The Mathworks Inc., USA). All reconstructed ^18^F-FDG PET images were spatially normalized to the Montreal Neurological Institute (MNI) reference space [21] using a standard brain ^18^F-FDG PET template provided with SPM2. Slices containing only the cerebellum were separated from these ^18^F-FDG PET images, normalized images of typical normal subjects were averaged to create a study-specific cerebellar template, and individual ^18^F-FDG PET images containing only cerebellar slices were normalized to the study-specific cerebellar template. The detailed procedure was described in our previous work [16]. ^18^F-FDG PET images showed that higher counts on the anatomically left cerebellar cortex were flipped to the contralateral side (right) during group analysis of asymmetry in cerebellar metabolism.

### VOI analysis

#### Definition of VOI

**Fig 1:**
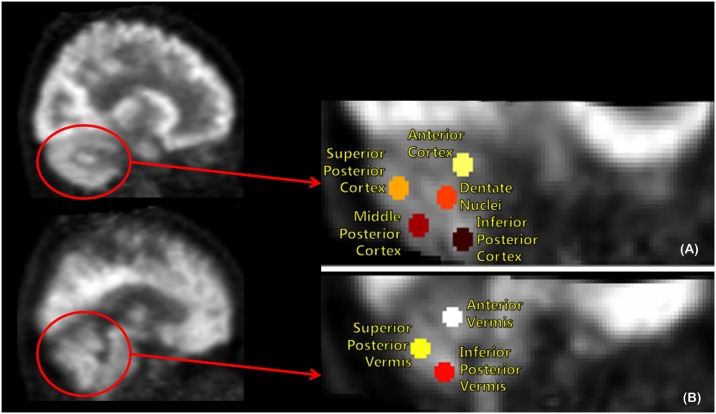
Volume of Interest (VOI) template on the cerebellum of a normal sagittal ^18^F-FDG PET image includes 13 VOIs in bilateral anterior cortex, superior/mid/inferior posterior cortex, dentate nuclei (A), anterior vermis, and superior/inferior posterior vermis (B).

Subregional cerebellar metabolic ratios were obtained from VOI values, which were defined using MRIcro^®^ version 1.40 (Chris Rorden, Columbia, SC, USA; www.mricro.com). Circular VOIs (diameter, 8 mm) were drawn manually on coregistered, spatially normalized, single T1-weighted magnetic resonance imaging (MRI) scans and ^18^F-FDG PET images of normal controls by an experienced nuclear medicine physician specializing in nuclear neurology (M.O). A 8-mm diameter circular VOI size was determined to reduce possible overestimation of metabolic activity due to spillover effects by adjacent structure [22]. VOIs for the cerebellum were defined as follows (Fig 1). The boundary between the anterior and posterior cerebellar lobes was visually determined by the primary fissure. Next, posterior cortex and posterior vermis were divided into three and two equal parts, respectively. As a result, cerebellar cortex was further divided into one anterior and three posterior lobar subregions. Bilateral anterior cortices (ANT) corresponded to anatomic lobules I–V, and superior/mid/inferior posterior cortices (SUPP/MIDP/INFP) corresponded to anatomic lobules VI–X. Cerebellar vermis was divided into anterior vermis (ANTV), and superior/inferior posterior vermis (SUPV/INFV). Dentate nuclei (DN), which were not clearly defined in T1-weighted MRI, were defined as regions with distinct metabolic activity in the middle of the cerebellar white matter (Fig 1).

#### Reference area for calculating metabolic ratio

To choose a reference area in voxelwise analysis, analysis of variance (ANOVA) with multiple comparison was used in SPM2, and robustness in age-related changes within cerebellar subregional and inter-subregional metabolic ratios were evaluated according to the reference areas (occipital/parietal cortex and whole gray matter). ^18^F-FDG PET counts were extracted from overlapping cerebral cortical areas defined by the sum of automated anatomical labeling (AAL) template VOIs and the normalized image of each subject.

#### Assessment of VOI values

The VOI template in the standard MNI space was automatically applied directly to the spatially normalized individual ^18^F-FDG PET images to extract VOI values using an in-house software program called ANTIQUE (AMC NM Toolkit for Image Quantification of Excellence), which was written in Interactive Data Language (version 8.2) [16]. First, we divided the mean VOI value of a specific cerebellar subregion by the mean VOI value of the reference area to calculate the metabolic ratio. We also calculated inter-subregional ratios, including anterior-posterior lobe ratio (APR) and asymmetric index (AI) using total cerebellar VOI values as follows:

$$Anterior\;to\;posterior\;lobe\;ratio\;\left(cortex\right)=\frac{\left[counts\;of\;ANT\right]}{\left[\;counts\;of\frac{\left(SUPP+MIDP+INFP\right)}{3}\right]}$$

$$Anterior\;to\;posterior\;lobe\;ratio\;\left(vermis\right)=\frac{\left[counts\;of\;ANTV\right]}{\left[counts\;of\;\frac{\left(SUPV+INFV\right)}{2}\right]}$$

$$\text{A}symmetric\;Index=\frac{\left[\sum counts\;of\;VOI\;in\;the\;right\;cerebellar\;hemisphere\right]-[\sum counts\;of\;VOI\;in\;the\;left\;cerebellar\;hemisphere]}{\left[\sum counts\;of\;VOI\;in\;the\;right\;cerebellar\;hemisphere\right]+[\sum counts\;of\;VOI\;in\;the\;left\;cerebellar\;hemisphere]}$$

### Voxelwise analysis

Spatial preprocessing and statistical analysis were performed using SPM2. After separation of slices containing the cerebellum, ^18^F-FDG PET images were spatially normalized to the study-specific cerebellar templates with a 12-parameter affine transformation. Spatially normalized images were smoothed by convolution with an isotropic Gaussian kernel of 12 mm FWHM to increase the signal-to-noise ratio [23]. Using the group comparison model in SPM2, we assessed differences in regional cerebellar glucose metabolism between patient groups and controls with a threshold of *p* < 0.01 (uncorrected) and an extent threshold of 100 voxels. Patient age was used as a confounding nuisance covariate during statistical analysis to minimize its impact. This statistical threshold was selected not only to capture a wide range of statistical differences but also to provide sufficient distinguishing power to identify separate clusters of metabolic differences.

### Statistical analysis

Descriptive statistics included means ± standard deviation or frequencies for each clinical characteristic. Comparisons of age at ^18^F-FDG PET examination and disease duration between healthy control and patient groups were performed using nonparametric statistics (Kruskal–Wallis H test) since some of the sample sizes were small and the assumption of a Gaussian distribution was not appropriate. Significance of intergroup differences was assessed by the Kruskal–Wallis test due to non-normal distribution. Post-hoc testing was used to identify statistically significant group differences, taking into account Bonferroni correction for multiple comparisons (*p* < 0.05/10).

Correlations between age, symptom duration, and regional VOI values and ratios were estimated by parametric Pearson's product-moment correlation coefficients in normal controls and patients with MSA-C and by nonparametric Spearman's rank correlation coefficients in patients with SCA type 2 or type 6. Data storage and analysis were performed using SPSS^®^ software (Statistical Package for the Social Science, version 18.0, Chicago, IL, USA).

## Results

### Reference area for calculating metabolic ratio

#### Voxelwise analysis

Voxelwise analysis by SPM after global count normalization indicated that parts of the occipital and parietal cortices showed little variance in regional metabolism among the disease groups and normal controls (S1 Fig).

#### Aging effect of metabolic ratio according to reference area

**Fig 2:**
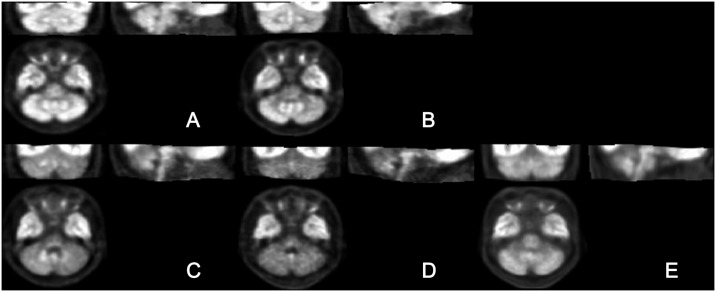
Representative images of spatially normalized ratios using study-specific templates of normal controls (A) and patients with crossed cerebellar diaschisis (B), multiple system atrophy of the cerebellar type (C), Spinocerebellar Ataxia (SCA) type 2 (D), and SCA type 6 (E).

Metabolic ratios of cerebellar subregions using occipital cortex as a reference for count normalization did not change with age (r = 0.141, *p* = 0.060 for ANT; r = −0.033, *p* = 0.665 for SUPP). While there was a weak positive correlation in ANT (r = 0.214, *p* = 0.004), there was no correlation in SUPP (r = 0.044, *p* = 0.556) by count normalization using parietal cortex as a reference. Moderate and weak positive correlations in ANT and SUPP, respectively, were observed by global count normalization (r = 0.448, *p* < 0.001 and r = 0.177, *p* = 0.018, respectively; S2 Fig). These results revealed that occipital cortex was appropriate for count normalization. Fig 2 shows representative images that were spatially normalized to the study-specific template and count normalization using occipital cortex.

### VOI analysis

#### Comparison of patients with healthy controls

**Fig 3:**
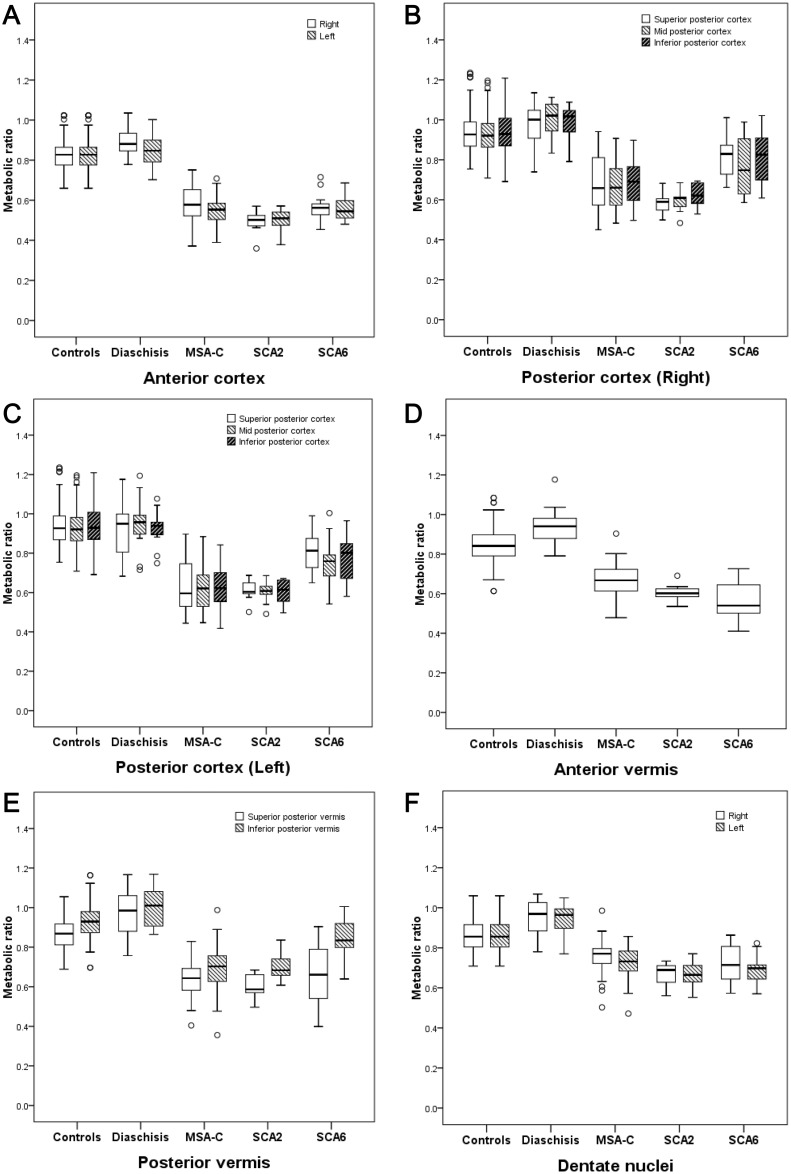
Subregional metabolic ratios in normal controls and disease groups. Metabolic ratios of anterior cortex (A), anterior vermis (B), right posterior cortex (C), left posterior cortex (D), posterior vermis (E), and dentate nuclei (F) were decreased in patients with multiple system atrophy of the cerebellar type (MSA-C) and spinocerebellar ataxia (SCA) type 2 and 6, compared to normal controls and those with crossed cerebellar diaschisis.

Patients with MSA-C and SCA type 2 showed severely decreased metabolic ratios in all cerebellar subregions compared to normal controls (*p* < 0.001 for all subregions, Fig 3). Patients with SCA type 6 showed decreased metabolic ratios in almost all cerebellar subregions (*p* < 0.001) except INFV (*p* = 0.006).

Analysis for anterior-posterior lobe ratio showed that patients with SCA type 2 (Right, 0.81 ± 0.05 vs. 0.88 ± 0.04, *p* < 0.001; Left, 0.83 ± 0.05 vs. 0.88 ± 0.04, *p* = 0.003) and SCA type 6 (Right, 0.72 ± 0.05 vs. 0.88 ± 0.04, *p <* 0.001; Left, 0.72 ± 0.05 vs. 0.88 ± 0.04, *p* < 0.001) exhibited lower ratios compared to normal controls, whereas no changes in anterior-posterior lobe ratios were observed in patients with CCD or MSA-C (Table 2).

**Table 2:** Anterior-posterior lobe ratios in cerebellar cortex and vermis, asymmetric indices of cortex and dentate nuclei, and their correlation with disease duration in controls, patients with Crossed Cerebellar Diaschisis (CCD), Multiple System Atrophy (MSA), and Spinocerebellar Atrophy (SCA) type 2 and type 6.

	Controls (n = 89)	Crossed cerebellar diaschisis (n = 15)	MSA-C (n = 44)			SCA2 (n = 9)			SCA6 (n = 14)		
	Mean ± SD	Mean ± SD	Mean ± SD	Correlation with duration^A^	*p*	Mean ± SD	Correlation with duration^A^	*p*	Mean ± SD	Correlation with duration^A^	*p*
Duration (year)			1.91± 1.76			6.78 ± 4.30			4.93 ± 4.80		
Asymmetric Index (Cortex)	0.02 ± 0.01	0.05 ± 0.03	0.05 ± 0.04	−0.169	0.278	0.01 ± 0.01	*−*0.118	0.763	0.02 ± 0.02	*−*0.206	0.479
Asymmetric Index (Dentate Nuclei)	0.02 ± 0.01	0.03 ± 0.01	0.03 ± 0.03	*−*0.064	0.683	0.01 ± 0.01	*−*0.294	0.442	0.03 ± 0.02	0.244	0.400
Anterior to posterior ratio (Right cortex)	0.88 ± 0.04	0.91 ± 0.06	0.87 ± 0.11	*−*0.252	0.103	0.81 ± 0.05	*−*0.765	0.016	0.72 ± 0.05	0.013	0.964
Anterior to posterior ratio (Left cortex)	0.88 ± 0.04	0.92 ± 0.09	0.88 ± 0.12	*−*0.372	0.014	0.83 ± 0.05	*−*0.143	0.714	0.72 ± 0.05	*−*0.335	0.241
Anterior to posterior ratio (Vermis)	0.94 ± 0.05	0.95 ± 0.04	1.01 ± 0.08	*−*0.043	0.784	0.92 ± 0.05	*−*0.605	0.084	0.75 ± 0.06	*−*0.340	0.235

Correlation with duration^A^: Correlation between disease duration and ratio was estimated using the parametric Pearson's product-moment correlation coefficient in normal controls and multiple system atrophy of the cerebellar type (MSA-C) group or the nonparametric Spearman's rank correlation coefficient in crossed cerebellar diaschisis (CCD), spinocerebellar atrophy (SCA) type 2, and SCA type 6.

However, analysis for asymmetry showed that patients with CCD (0.05 ± 0.03 vs. 0.02 ± 0.01, *p* < 0.001) and MSA-C (0.05 ± 0.04 vs. 0.02 ± 0.01, *p* < 0.001) exhibited highly asymmetric indices compared to normal controls, which was not observed in patients with SCA type 2 or 6.

#### Comparison among SCA type 2, SCA type 6, and MSA-C

A post hoc analysis revealed that metabolic ratios in patients with SCA type 2, SCA type 6, MSA-C showed different cerebellar metabolism from each other in several subregions of the cerebellum.

There were significant reductions in metabolic ratios in right DN (0.69 ± 0.06 vs. 0.78 ± 0.08, *p* < 0.001) and right ANT (0.50 ± 0.06 vs. 0.58 ± 0.08, *p* = 0.001) of patients with SCA type 2 compared to those of patients with MSA-C. There were no significant differences in anterior-posterior lobe metabolic ratios between SCA type 2 and MSA-C in cerebellar cortex (Right, 0.81 ± 0.05 vs. 0.87 ± 0.11, *p* = 0.255; Left, 0.83 ± 0.05 vs. 0.88 ± 0.12, *p* = 0.236), which was significantly different in vermis between the SCA type 2 and MSA-C groups (0.92 ± 0.05 vs. 1.01 ± 0.08, respectively, *p* = 0.001). Metabolic ratios were more asymmetric in patients with MSA-C than in those with SCA type 2 (0.05 ± 0.04 vs. 0.01 ± 0.01, *p* = 0.001).

Compared to patients with MSA-C, patients with SCA type 6 showed higher metabolic ratios in both SUPPs (Right, 0.83 ± 0.11 vs. 0.69 ± 0.14, *p* = 0.002; Left, 0.81 ± 0.10 vs. 0.63 ± 0.13, *p* < 0.001), left MIDP (0.75 ± 0.12 vs. 0.63 ± 0.12, *p* = 0.003), left INFP (0.78 ± 0.11 vs. 0.64 ± 0.10, *p* < 0.001), and INFV (0.82 ± 0.11 vs. 0.73 ± 0.11, *p* < 0.001). The metabolic ratios in ANTV were lower in patients with SCA type 6 (0.57 ± 0.10 vs. 0.71 ± 0.08, *p* = 0.001) than in patients with MSA-C. Anterior-posterior lobe ratios in cortex were lower in patients with SCA type 6 (Right, 0.72 ± 0.05 vs. 0.87 ± 0.11, *p* = 0.001; Left, 0.72 ± 0.05 vs. 0.88 ± 0.12, *p* = 0.001) than in those with MSA-C. MSA-C was more asymmetric than SCA type 6 (0.05 ± 0.04 vs. 0.02 ± 0.02, *p* = 0.012).

Patients with SCA type 6 demonstrated higher metabolic ratios in both SUPPs (Right, 0.83 ± 0.11 vs. 0.61 ± 0.05, *p* < 0.001; Left, 0.81 ± 0.10 vs. 0.63 ± 0.06, *p* < 0.001), MIDP (Right, 0.77 ± 0.14 vs. 0.63 ± 0.06, *p* = 0.002; Left, 0.75 ± 0.12 vs. 0.64 ± 0.06, *p* = 0.003), and INFP (Right, 0.81 ± 0.13 vs. 0.63 ± 0.06, *p* = 0.002; Left, 0.78 ± 0.12 vs. 0.60 ± 0.13, *p* = 0.001) than patients with SCA type 2. Anterior- posterior lobe ratios were lower in patients with SCA type 6 (Right, 0.72 ± 0.05 vs. 0.81 ± 0.05, *p* = 0.002; Left, 0.72 ± 0.05 vs. 0.83 ± 0.05, *p* = 0.001; Vermis, 0.75 ± 0.06 vs. 0.92 ± 0.05, *p <* 0.001) than in patients with SCA type 2. Neither SCA type 2 nor SCA type 6 demonstrated asymmetry (0.01 ± 0.01 vs. 0.02 ± 0.02, *p* = 0.166).

#### Metabolic ratio with age

There were no correlations between age and metabolic ratios in anterior (r = 0.141, *p* = 0.060) or posterior (r = −0.033 *p* = 0.665) lobes in normal controls or in patients with MSA-C (anterior lobe, r = −0.104, *p* = 0.503; posterior lobe, r = −0.122, *p* = 0.431). There was a weak positive correlation between age and anterior-posterior lobe ratio (r = 0.158, *p* = 0.035) in normal controls, whereas such correlation was not observed in MSA-C (r = 0.053, *p* = 0.734), SCA type 2, or SCA type 6.

#### Inter-subregional ratios in relation with symptom duration

In MSA-C, there was no correlation between symptom duration and asymmetry. Anterior-posterior lobe ratio showed a weak negative correlation with symptom duration (r = −0.372, *p* = 0.014) in left cortex.

In SCA type 2, there was no correlation between symptom duration and asymmetry. Anterior-posterior lobe ratio showed a strong negative correlation with symptom duration (r = −0.765, *p* = 0.016) in right cortex.

In SCA type 6, there were no correlations between duration and inter-subregional ratios.

### Voxelwise analyses

#### Comparison of patients with normal controls

**Fig 4:**
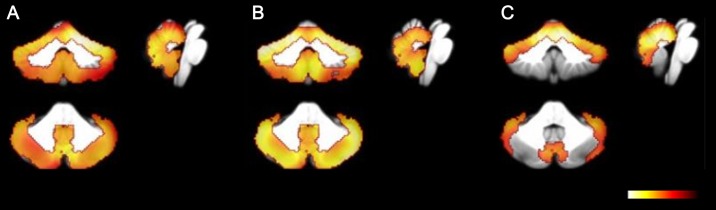
Coronal, sagittal, and transverse statistical parametric mapping images in patients with ataxia compared to normal controls (*p* < 0.001, FWE). Patients with multiple system atrophy of the cerebellar type (MSA-C, A) and spinocerebellar ataxia (SCA) type 2 (B) show decreased metabolism of the entire cerebellum. Patients with SCA type 6 (C) show decreased metabolism mainly in the anterior and superior posterior lobes of the cerebellum.

Both MSA-C and SCA type 2 groups showed significantly decreased metabolic ratios in whole cerebellar cortex and vermis, compared with normal controls. Additionally, patients with MSA-C exhibited a more asymmetric pattern compared to those with SCA type 2. Patients with SCA type 6 showed significantly decreased metabolic ratios mainly in the anterior and superior posterior lobes (Fig 4).

#### Comparison among disease groups

The decrease in metabolic ratios in the right anterior and left posterior lobes was more pronounced in patients with SCA type 2 than in those with MSA-C. Additionally, the decrease in metabolic ratios in the right anterior lobe of patients with SCA type 6 was more than that found in patients with MSA-C. These findings reflected the relatively more pronounced asymmetric nature of metabolic patterns observed in MSA-C compared to the SCA subtypes. Comparison between SCA subtypes revealed that the decrease in metabolic ratios of the posterior cerebellar lobes was more marked in patients with SCA type 2 than in those with SCA type 6 (S3 Fig).

## Discussion

To our knowledge, the present study includes the most comprehensive and detailed subregional analysis of cerebellar metabolism using ^18^F-FDG PET to date. Specifically, we observed that cerebellar metabolism in the anterior lobe was relatively decreased compared to posterior lobe in patients with cerebellar ataxia regardless of etiology, in contrast to normal controls and patients with CCD. This observation was not affected by age, which supported distinct underlying pathophysiologies leading to cerebellar degeneration. Anterior-posterior lobe uptake ratios showed variable degrees of negative correlation with disease progression in patients with MSA-C and SCA type 2, which revealed the preferential involvement of anterior lobe, whereas asymmetry in none of the patient groups were affected by disease duration.

Anterior cerebellar lobe, functionally corresponding to spinocerebellum, plays a key role in maintaining posture, muscle tone, and gait [19, 24]. The cardinal feature of spinocerebellar disease is involvement of lower extremities resulting in wide-based ataxic gait with small hesitant steps [19, 25]. Therefore, reduced uptake in the anterior cerebellar lobe of patients with cerebellar ataxia is a logical consequence of the disruption in motor functions executed by spinocerebellum.

Although decreased metabolism in anterior lobe is common among patients with cerebellar ataxia, characteristic patterns of cerebellar subregional metabolism were distinct in each etiology. In patients with MSA-C, metabolic alterations were observed not only in the anterior-posterior ratio, which was consistent with the results of previous reports [13], but also in patterns of asymmetry. Tendency for asymmetry in cerebellar metabolism was a distinctive pattern of MSA-C among patients with cerebellar ataxia in this study and mirrored the asymmetry in clinical features of patients with probable MSA-C determined by diagnostic criteria [26]. These motor findings are often asymmetrical during the natural course of disease and may be pronounced [27]. Furthermore, about 50% of probable MSA patients reported asymmetric onset of symptoms [28]. Dopamine transporter (DAT) imaging of patients with a definitive postmortem diagnosis also showed a trend toward greater asymmetry of striatal DAT binding in MSA than in PD [29]. Although decreased cerebellar glucose metabolism in bilateral hemispheres and vermis is well known [13, 15, 30], its asymmetrical pattern was unnoticed. Excluding lateralization of individual ^18^F-FDG PET images before group comparisons might result in neutralization of asymmetric trends. Considering the relatively short duration between onset of symptoms and ^18^F-FDG PET imaging in this study, the asymmetric patterns of cerebellar metabolism could be a diagnostic clue in early stage of disease. Reaching milestones after disease onset takes more time, usually 20 to 30 months, in patients with MSA-C [31].

There were also some differences in subregional hypometabolism patterns between SCA type 2 and 6, despite similar patterns of preferential decreases in metabolic ratios in the anterior lobe of cerebellum. Patients with SCA type 2 showed the most extensive hypometabolism patterns in cerebellum. To our knowledge, only a few studies assessed cerebellar metabolism in SCA type 2 using ^18^F-FDG PET. Inagaki *et al*. first reported decreased cerebellar metabolism in two symptomatic and three asymptomatic individuals who were genetically confirmed as SCA type 2 [14]. Regional patterns of cerebral glucose metabolism by voxel-based ^18^F-FDG PET analysis showed that cerebellar metabolism was most severely compromised in SCA type 2, compared with SCA types 3 and 6 [32]. This was consistent with pathologic findings of marked loss of Purkinje cells in cerebellar cortex as well as the loss of myelinated fibers with gliosis in inferior and middle cerebellar peduncles, cerebellar white matter, and fasciculus cuneatus [33]. No reports comparing the differences in cerebellar metabolism of patients with MSA-C and SCA type 2 are currently available.

Patients with SCA type 6 exhibited hypometabolism mainly in the anterior lobe of cerebellar cortex and vermis, with relative preservation of the posterior lobe including inferior vermis. This finding is concordant with preferential superior vermian atrophy with relative sparing of inferior vermis in SCA type 6 reported in morphological and neuropathological studies [34, 35]. While widespread reduction in glucose metabolism of cerebral cortex, basal ganglia, and cerebellum were previously reported in patients with SCA type 6 compared with healthy controls [36], no regional analyses were performed. Compared with SCA type 2, SCA type 6 demonstrated a lesser degree and extent of cerebellar hypometabolism [32], which were consistent with the results of the present study. Clinically, this form of SCA is characterized as pure cerebellar ataxia and can be accompanied by dysarthria, nystagmus, dysphagia, and even loss of proprioception and dystonia [37–39]. The pathology includes mainly the degeneration of Purkinje cells in cerebellar cortex, leaving dentate nucleus, deep cerebellar white matter, and cerebellar peduncles unaffected [39]. And the grossly normal cerebellum in some patients with SCA type 6 showed loss of Purkinje cells, being more marked in the superior vermis suggesting that the initial and primary change occurs in the cerebellar Purkinje cells, starting from the superior vermis [35].

In summary, distinct cerebellar subregional metabolic alterations among different types of cerebellar ataxia were attributable to the distinctive neuropathological changes in cerebellum, as reported in previous studies with acetylcholinesterase and DAT PET [40, 41]. In contrast, patients with CCD, characterized by functional impairment in a region far from the site of a brain lesion that is anatomically connected by fiber tracts [42], showed relatively preserved metabolism in the anterior lobe resulting in high anterior-posterior lobe ratio. As the majority of patients with CCD in this study had brain tumors in the frontal lobe, this finding reflected alterations in the corticopontocerebellar tract passing through the anterior limb of internal capsule to ipsilateral pontine nuclei and crossing the midline via the middle cerebellar peduncle [43, 44]. This finding was supported by anatomical observations of Brodal *et al*. who demonstrated that the majority of corticopontocerebellar fibers arose from the frontal cortex [45]. Brain tumors located in locations other than the frontal cortex would disrupt the corticopontocerebellar tract to a lesser degree, leading to different patterns in patients with CCD [46].

Choosing the occipital cortex as a reference area was another major outcome of this study. Correct identification of a reference area is an important prerequisite for accurate semi-quantitative analysis of cerebellar subregional metabolism. For count normalization, the most widely used normalization method computes the ratio of voxel value to the mean glucose metabolism of all voxels within a reference region, commonly defined as the template of the entire gray matter [47]. Certain diseases causing altered cerebellar metabolism, however, also involve the cerebral cortex [48], which makes the detection of statistically significant group differences difficult [49, 50]. Although areas affected by disease are different in their anatomical distribution [51], performing a count normalization procedure is necessary to compare relative regional metabolic ratios between healthy controls or different diagnoses. This arithmetic transformation, termed normalization, is often utilized to reduce variation and allows for the more sensitive detection of disease-dependent patterns of altered metabolism [52]. Therefore, choosing a certain supratentorial area as a reference region rather than the entire cerebral cortex for normalization is more appropriate for the assessment of cerebellar metabolism.

As emphasized by Yakushev *et al*. [53], in patients as well as in healthy controls, appropriate reference areas should be maximally stable, minimally susceptible to external physiological stimuli, unaffected by the disease of interest, and reliable and easy to determine. The importance of valid reference regions for differential diagnosis is well established in diseases involving cerebral cortex such as dementia [53, 54]. Up to date, only a few studies investigated altered cerebellar subregional metabolism; thus, there is no consensus on optimal supratentorial areas for count normalization of cerebellar uptake ratios in patients with cerebellar ataxia. In previous studies, variable reference areas such as frontal cortex, thalamus, and whole gray matter were utilized [14, 55–58]. While these regions all succeeded in showing decreased cerebellar uptake ratios, patients were compared only with normal controls. Well-known age-related metabolic decline in frontal cortex and global mean [52, 59] suggests that this approach might be inappropriate for reference. These caveats could mask subtle differences between distinct types of cerebellar ataxia in this study.

At the start of the present study, choosing a specific supratentorial region as a reference for count normalization presented a challenge as previous reports indicated that not only cerebellar but also cerebral regions were involved in SCA as determined by ^18^F-FDG PET [36, 40, 48]. Thus, we choose an area with minimal changes among disease groups and controls using voxelwise analysis. This method guaranteed a consistent measurement of metabolic rate at least in patients and subjects included this study. As shown in our results, compared with global count normalization, count normalization with occipital or parietal cortex resulted in more robust aging effects; this was consistent with the results of a previous ^18^F-FDG PET study showing that parietal cortex was relatively spared in cerebellar ataxia [60]. Thus, the aging effect observed in global count normalization was largely due to the well-known age-related decline in frontal metabolism as mentioned above [61].

The present study has several major limitations. First, due to the lack of clinical parameters such as the International Cooperative Ataxia Rating Scale (ICARS) [62] and the Scale for Assessment and Rating of Ataxia (SARA) [63] scores in the medical records of patients with cerebellar ataxia, we could not correlate clinical severity with cerebellar subregional metabolism. Further studies are needed to investigate the correlation between the severity of ataxia and cerebellar subregional metabolism, which will provide valuable information on disease progression and metabolic deterioration. Second, we could not completely separate cerebellum from other structures of the brain. On voxelwise analysis, some areas with different metabolic ratios were located outside the cerebellum, which were not included in the design of the present study. Furthermore, certain areas with minimum detectable differences in the cerebellum might be overlooked. Third, patients with MSA-C in the present study were diagnosed clinically, and a possible diagnosis of SCA was ruled out based on a set of genetic tests that included only the common SCA types at our institution. Therefore, misdiagnoses could not be ruled out in the absence of postmortem verification [8]. Although all diagnoses were based on strict diagnostic criteria, a longer period of follow-up is needed. Finally, we only focused on altered metabolic patterns in cerebellum and acknowledge the need for further studies designed to assess functional topography of cerebellar and cerebral connections.

Despite these limitations, the detailed subregional analysis of cerebellar metabolism in our study provided insights into the different pathophysiological processes not only among ataxic patients but also in other etiologies of cerebellar metabolic alterations.

## Conclusions

Quantitative analysis of cerebellar subregional metabolism by ^18^F-FDG PET revealed the preferential involvement of anterior lobe in patients with cerebellar ataxia, which corresponded to spinocerebellum that controls balance of gait, whereas patients with CCD showed more asymmetric and preferential involvement of posterior lobe. These results might reflect the functionally and metabolically distinct involvement of cerebellar subregions among cerebellar diseases.

## Supporting information

S1 Fig Choice of reference area among cerebral cortices for the calculation of cerebellar metabolic ratio. Voxelwise analysis of the disease and control groups (except for crossed cerebellar diaschisis) shows little variance among the groups in parts of the parietal and occipital cortices.
(TIF)

S2 Fig Age-related changes in metabolic ratios of the Anterior (ANT, Figure A in S2 File) and Superior Posterior (SUPP, Figure B in S2 File) lobes according to reference areas in normal controls. Metabolic ratios of cerebellar subregions with occipital cortex used as reference for count normalization did not change with age (r = 0.141, *p* = 0.060 for ANT; r = −0.033, *p* = 0.665 for SUPP) (Right, Figure A and B in S2 File). There was a weak positive correlation in ANT (r = 0.214, *p* = 0.004) but no correlation in SUPP (r = 0.044, *p* = 0.556) by count normalization with parietal cortex used as reference (Middle, Figure A and B in S2 File). Moderate and weak positive correlations in ANT and SUPP, respectively, were observed by global count normalization (r = 0.448, *p* < 0.001 for ANT; r = 0.177, *p* = 0.018 for SUPP) (Left, Figure A and B in S2 File).
(TIF)

S3 Fig Coronal, sagittal, and transverse statistical parametric mapping images of comparisons among disease groups (*p* < 0.01, uncorrected). Patients with spinocerebellar ataxia SCA type 2 (Figure A in S3 File) show decreased metabolism in the right anterior and left posterior lobes, compared to those in patients with multiple system atrophy of the cerebellar type (MSA-C). Patients with SCA type 6 (Figure B in S3 File) shows decreased metabolism in the anterior lobe and anterior vermis compared to those in patients with MSA-C. Patients with SCA type 2 (Figure C in S3 File) shows decreased metabolism in the posterior lobe compared to that in patients with SCA type 6.
(TIF)

## References

[1] Abele M, Burk K, Schols L, Schwartz S, Besenthal I, Dichgans J, et al The aetiology of sporadic adult-onset ataxia. Brain. 2002; 125: 961–968. 11960886 10.1093/brain/awf107

[2] Klockgether T. Sporadic ataxia with adult onset: classification and diagnostic criteria. Lancet Neurol. 2010; 9: 94–104. 10.1016/S1474-4422(09)70305-9 20083040

[3] Wenning GK, Geser F. Multiple system atrophy. Rev Neurol (Paris). 2003; 159: 3S31–38. 12773886

[4] Koga S, Aoki N, Uitti RJ, van Gerpen JA, Cheshire WP, Josephs KA, et al When DLB, PD, and PSP masquerade as MSA An autopsy study of 134 patients. Neurology. 2015; 85: 404–412. 10.1212/WNL.0000000000001807 26138942 PMC4534078

[5] Hughes AJ, Daniel SE, Ben-Shlomo Y, Lees AJ. The accuracy of diagnosis of parkinsonian syndromes in a specialist movement disorder service. Brain. 2002; 125: 861–870. 11912118 10.1093/brain/awf080

[6] Kim HJ, Jeon BS, Shin J, Lee WW, Park H, Jung YJ, et al Should genetic testing for SCAs be included in the diagnostic workup for MSA? Neurology. 2014; 83: 1733–1738. 10.1212/WNL.0000000000000965 25298309

[7] Muzaimi MB, Thomas J, Palmer-Smith S, Rosser L, Harper PS, Wiles CM, et al Population based study of late onset cerebellar ataxia in south east Wales. J Neurol Neurosurg Psychiatry. 2004; 75: 1129–1134. 10.1136/jnnp.2003.014662 15258214 PMC1739172

[8] Litvan I, Goetz CG, Jankovic J, Wenning GK, Booth V, Bartko JJ, et al What is the accuracy of the clinical diagnosis of multiple system atrophy? a clinicopathologic study. Arch Neurol. 1997; 54: 937–944. 9267967 10.1001/archneur.1997.00550200007003

[9] Yagishita S, Inoue M. Clinicopathology of spinocerebellar degeneration: its correlation to the unstable CAG repeat of the affected gene. Pathol Int. 1997; 47: 1–15. 9051687 10.1111/j.1440-1827.1997.tb04429.x

[10] Wenning GK, Tison F, Ben Shlomo Y, Daniel SE, Quinn NP. Multiple system atrophy: a review of 203 pathologically proven cases. Mov Disord. 1997; 12: 133–147. 10.1002/mds.870120203 9087971

[11] Maas RP, van Gaalen J, Klockgether T, van de Warrenburg BP. The preclinical stage of spinocerebellar ataxias. Neurology. 2015; 85: 96–103. 10.1212/WNL.0000000000001711 26062625

[12] Brockmann K, Reimold M, Globas C, Hauser TK, Walter U, Machulla HJ, et al PET and MRI reveal early evidence of neurodegeneration in spinocerebellar ataxia type 17. J Nucl Med. 2012; 53: 1074–1080. 10.2967/jnumed.111.101543 22653791

[13] Lyoo CH, Jeong Y, Ryu YH, Lee SY, Song TJ, Lee JH, et al Effects of disease duration on the clinical features and brain glucose metabolism in patients with mixed type multiple system atrophy. Brain. 2008; 131: 438–446. 10.1093/brain/awm328 18178568

[14] Inagaki A, Iida A, Matsubara M, Inagaki H. Positron emission tomography and magnetic resonance imaging in spinocerebellar ataxia type 2: a study of symptomatic and asymptomatic individuals. Eur J Neurol. 2005; 12: 725–728. 10.1111/j.1468-1331.2005.01011.x 16128876

[15] Eckert T, Barnes A, Dhawan V, Frucht S, Gordon MF, Feigin AS, et al FDG PET in the differential diagnosis of parkinsonian disorders. NeuroImage. 2005; 26: 912–921. 10.1016/j.neuroimage.2005.03.012 15955501

[16] Oh JS, Oh M, Chung SJ, Kim JS. Cerebellum-specific 18F-FDG PET analysis for the detection of subregional glucose metabolism changes in spinocerebellar ataxia. Neuroreport. 2014; 25: 1198–1202. 10.1097/WNR.0000000000000247 25144395

[17] Makris N, Schlerf JE, Hodge SM, Haselgrove C, Albaugh MD, Seidman LJ, et al MRI-based surface-assisted parcellation of human cerebellar cortex: an anatomically specified method with estimate of reliability. NeuroImage. 2005; 25: 1146–1160. 10.1016/j.neuroimage.2004.12.056 15850732

[18] Roostaei T, Nazeri A, Sahraian MA, Minagar A. The human cerebellum: a review of physiologic neuroanatomy. Neurol Clin. 2014; 32: 859–869. 10.1016/j.ncl.2014.07.013 25439284

[19] Mauritz KH, Dichgans J, Hufschmidt A. Quantitative analysis of stance in late cortical cerebellar atrophy of the anterior lobe and other forms of cerebellar ataxia. Brain. 1979; 102: 461–482. 315255 10.1093/brain/102.3.461

[20] Gilman S, Wenning GK, Low PA, Brooks DJ, Mathias CJ, Trojanowski JQ, et al Second consensus statement on the diagnosis of multiple system atrophy. Neurology. 2008; 71: 670–676. 10.1212/01.wnl.0000324625.00404.15 18725592 PMC2676993

[21] Evans AC, Collins DL, Mills SR, Brown ED, Kelly RL, Peters TM. 3D statistical neuroanatomical models from 305 MRI volumes. In: IEEE Nuclear and Plasma Sciences Society, editor. Nuclear science symposium and medical imaging conference: 1993 IEEE conference record. 3. San Francisco, CA: IEEE; 1993. pp. 1813–1817.

[22] Rousset OG, Ma Y, Evans AC. Correction for partial volume effects in PET: principle and validation. The Journal of Nuclear Medicine. 1998; 39: 904 9591599

[23] Talairach J, Tournoux P. Co-planar stereotaxic atlas of the human brain 3-Dimensional proportional system: an approach to cerebral imaging Stuttgart: Thieme; 1988.

[24] Oscarsson O. Functional organization of the spino- and cuneocerebellar tracts. Physiol Rev. 1965; 45: 495–522. 14337566 10.1152/physrev.1965.45.3.495

[25] Fredericks CM. Disorders of the cerebellum and its connections In: Fredericks CM, Saladin LK, Fredericks CM, editors. Pathophysiology of the motor systems: principles and clinical presentations. Philadelphia: F. A. Davis Company; 1996 pp. 445–466.

[26] Wenning GK, Ben Shlomo Y, Magalhaes M, Daniel SE, Quinn NP. Clinical features and natural history of multiple system atrophy: an analysis of 100 cases. Brain. 1994; 117 (Pt 4): 835–845. 7922469 10.1093/brain/117.4.835

[27] Batla A, Stamelou M, Mensikova K, Kaiserova M, Tuckova L, Kanovsky P, et al Markedly asymmetric presentation in multiple system atrophy. Parkinsonism Relat Disord. 2013; 19: 901–905. 10.1016/j.parkreldis.2013.05.004 23746453

[28] Wullner U, Schmitz-Hubsch T, Abele M, Antony G, Bauer P, Eggert K. Features of probable multiple system atrophy patients identified among 4770 patients with parkinsonism enrolled in the multicentre registry of the German Competence Network on Parkinson's disease. J Neural Transm (Vienna). 2007; 114: 1161–1165. 17510732 10.1007/s00702-007-0746-0

[29] Perju-Dumbrava LD, Kovacs GG, Pirker S, Jellinger K, Hoffmann M, Asenbaum S, et al Dopamine transporter imaging in autopsy-confirmed Parkinson's disease and multiple system atrophy. Mov Disord. 2012; 27: 65–71. 10.1002/mds.24000 22102521

[30] Tang CC, Poston KL, Eckert T, Feigin A, Frucht S, Gudesblatt M, et al Differential diagnosis of parkinsonism: a metabolic imaging study using pattern analysis. Lancet Neurol. 2010; 9: 149–158. 10.1016/S1474-4422(10)70002-8 20061183 PMC4617666

[31] Lee SW, Koh SB. Clinical features and disability milestones in multiple system atrophy and progressive supranuclear palsy. J Mov Disord. 2012; 5: 42–47. 10.14802/jmd.12010 24868413 PMC4027659

[32] Wang PS, Liu RS, Yang BH, Soong BW. Regional patterns of cerebral glucose metabolism in spinocerebellar ataxia type 2, 3 and 6: a voxel-based FDG-positron emission tomography analysis. J Neurol. 2007; 254: 838–845. 10.1007/s00415-006-0383-9 17468965

[33] Durr A, Smadja D, Cancel G, Lezin A, Stevanin G, Mikol J, et al Autosomal dominant cerebellar ataxia type I in Martinique (French West Indies): clinical and neuropathological analysis of 53 patients from three unrelated SCA2 families. Brain. 1995; 118(Pt 6): 1573–1581. 8595486 10.1093/brain/118.6.1573

[34] Butteriss D, Chinnery P, Birchall D. Radiological characterization of spinocerebellar ataxia type 6. Br J Radiol. 2005; 78: 694–696. 10.1259/bjr/73834093 16046419

[35] Takahashi H, Ikeuchi T, Honma Y, Hayashi S, Tsuji S. Autosomal dominant cerebellar ataxia (SCA6): clinical, genetic and neuropathological study in a family. Acta Neuropathol. 1998; 95: 333–337. 9560009 10.1007/s004010050807

[36] Soong B, Liu R, Wu L, Lu Y, Lee H. Metabolic characterization of spinocerebellar ataxia type 6. Arch Neurol. 2001; 58: 300–304. 11176970 10.1001/archneur.58.2.300

[37] Stevanin G, Durr A, David G, Didierjean O, Cancel G, Rivaud S, et al Clinical and molecular features of spinocerebellar ataxia type 6. Neurology. 1997; 49: 1243–1246. 9371901 10.1212/wnl.49.5.1243

[38] Matsumura R, Futamura N, Fujimoto Y, Yanagimoto S, Horikawa H, Suzumura A, et al Spinocerebellar ataxia type 6. Molecular and clinical features of 35 Japanese patients including one homozygous for the CAG repeat expansion. Neurology. 1997; 49: 1238–1243. 9371900 10.1212/wnl.49.5.1238

[39] Gomez CM, Thompson RM, Gammack JT, Perlman SL, Dobyns WB, Truwit CL, et al Spinocerebellar ataxia type 6: gaze-evoked and vertical nystagmus, Purkinje cell degeneration, and variable age of onset. Ann Neurol. 1997; 42: 933–950. 10.1002/ana.410420616 9403487

[40] Hirano S, Shinotoh H, Arai K, Aotsuka A, Yasuno F, Tanaka N, et al PET study of brain acetylcholinesterase in cerebellar degenerative disorders. Mov Disord. 2008; 23: 1154–1160. 10.1002/mds.22056 18412283

[41] Wullner U, Reimold M, Abele M, Burk K, Minnerop M, Dohmen BM, et al Dopamine transporter positron emission tomography in spinocerebellar ataxias type 1, 2, 3, and 6. Arch Neurol. 2005; 62: 1280–1285. 10.1001/archneur.62.8.1280 16087769

[42] Pantano P, Baron JC, Samson Y, Bousser MG, Derouesne C, Comar D. Crossed cerebellar diaschisis. Further studies. Brain. 1986; 109 (Pt 4): 677–694. 3488093 10.1093/brain/109.4.677

[43] Diedrichsen J, Balsters JH, Flavell J, Cussans E, Ramnani N. A probabilistic MR atlas of the human cerebellum. NeuroImage. 2009; 46: 39–46. 10.1016/j.neuroimage.2009.01.045 19457380

[44] Kim J, Lee SK, Lee JD, Kim YW, Kim DI. Decreased fractional anisotropy of middle cerebellar peduncle in crossed cerebellar diaschisis: diffusion-tensor imaging-positron-emission tomography correlation study. AJNR Am J Neuroradiol. 2005; 26: 2224–2228. 16219826 PMC7976157

[45] Brodal A. Cerebrocerebellar pathways. Anatomical data and some functional implications. Acta Neurol Scand Suppl. 1972; 51: 153–195. 4351192

[46] Otte A, Roelcke U, von Ammon K, Hausmann O, Maguire RP, Missimer J, et al Crossed cerebellar diaschisis and brain tumor biochemistry studied with positron emission tomography, [18F]fluorodeoxyglucose and [11C]methionine. J Neurol Sci. 1998; 156: 73–77. 9559990 10.1016/s0022-510x(98)00019-7

[47] Kushner M, Tobin M, Alavi A, Chawluk J, Rosen M, Fazekas F, et al Cerebellar glucose consumption in normal and pathologic states using fluorine-FDG and PET. J Nucl Med. 1987; 28: 1667–1670. 3499490

[48] Harris DA, Briere RA, Cheu E, Makoff G, McFarland KS, Roodman A, et al Limit on the branching ratio of KL—> pi 0 micro+ micro. Phys Rev Lett. 1993; 71: 3914–3917. 10.1103/PhysRevLett.71.3914 10055107

[49] Diamant M, Harms MP, Immink RV, Van Lieshout JJ, Van Montfrans GA. Twenty-four-hour non-invasive monitoring of systemic haemodynamics and cerebral blood flow velocity in healthy humans. Acta Physiol Scand. 2002; 175: 1–9. 10.1046/j.1365-201X.2002.00953.x 11982498

[50] Kuhl DE, Metter EJ, Riege WH, Phelps ME. Effects of human aging on patterns of local cerebral glucose utilization determined by the [18F]fluorodeoxyglucose method. J Cereb Blood Flow Metab. 1982; 2: 163–171. 10.1038/jcbfm.1982.15 6978885

[51] Metter EJ, Kempler D, Jackson CA, Hanson WR, Riege WH, Camras LR, et al Cerebellar glucose metabolism in chronic aphasia. Neurology. 1987; 37: 1599–1606. 3658163 10.1212/wnl.37.10.1599

[52] Borghammer P, Jonsdottir KY, Cumming P, Ostergaard K, Vang K, Ashkanian M, et al Normalization in PET group comparison studies—the importance of a valid reference region. NeuroImage. 2008; 40: 529–540. 10.1016/j.neuroimage.2007.12.057 18258457

[53] Yakushev I, Landvogt C, Buchholz HG, Fellgiebel A, Hammers A, Scheurich A, et al Choice of reference area in studies of Alzheimer's disease using positron emission tomography with fluorodeoxyglucose-F18. Psychiatry Res. 2008; 164: 143–153. 10.1016/j.pscychresns.2007.11.004 18930634

[54] Dukart J, Mueller K, Horstmann A, Vogt B, Frisch S, Barthel H, et al Differential effects of global and cerebellar normalization on detection and differentiation of dementia in FDG-PET studies. NeuroImage. 2010; 49: 1490–1495. 10.1016/j.neuroimage.2009.09.017 19770055

[55] Soong BW, Liu RS. Positron emission tomography in asymptomatic gene carriers of Machado-Joseph disease. J Neurol Neurosurg Psychiatry. 1998; 64: 499–504. 9576542 10.1136/jnnp.64.4.499 PMC2170028

[56] Gilman S, Markel DS, Koeppe RA, Junck L, Kluin KJ, Gebarski SS, et al Cerebellar and brainstem hypometabolism in olivopontocerebellar atrophy detected with positron emission tomography. Ann Neurol. 1988; 23: 223–230. 10.1002/ana.410230303 3259853

[57] Mishina M, Senda M, Ishii K, Ohyama M, Kitamura S, Katayama Y. Cerebellar activation during ataxic gait in olivopontocerebellar atrophy: a PET study. Acta Neurol Scand. 1999; 100: 369–376. 10589796 10.1111/j.1600-0404.1999.tb01055.x

[58] Matthew E, Nordahl T, Schut L, King AC, Cohen R. Metabolic and cognitive changes in hereditary ataxia. J Neurol Sci. 1993; 119: 134–140. 8277326 10.1016/0022-510x(93)90125-i

[59] Tumeh PC, Alavi A, Houseni M, Greenfield A, Chryssikos T, Newberg A, et al Structural and functional imaging correlates for age-related changes in the brain. Semin Nucl Med. 2007; 37: 69–87. 10.1053/j.semnuclmed.2006.10.002 17289456

[60] Rudolf J, Grond M, Hilker R, Ghaemi M, Jacobs A, Heiss W. Relative sparing of the parietal cortex in cerebellar ataxia documented by positron emission tomography. Clin Neurol Neurosurg. 2000; 102: 210–214. 11154806 10.1016/s0303-8467(00)00113-x

[61] Volkow ND, Logan J, Fowler JS, Wang GJ, Gur RC, Wong C, et al Association between age-related decline in brain dopamine activity and impairment in frontal and cingulate metabolism. Am J Psychiatry. 2000; 157: 75–80. 10.1176/ajp.157.1.75 10618016

[62] Schmitz-Hubsch T, du Montcel ST, Baliko L, Berciano J, Boesch S, Depondt C, et al Scale for the assessment and rating of ataxia: development of a new clinical scale. Neurology. 2006; 66: 1717–1720. 10.1212/01.wnl.0000219042.60538.92 16769946

[63] Yabe I, Matsushima M, Soma H, Basri R, Sasaki H. Usefulness of the scale for assessment and rating of ataxia (SARA). J Neurol Sci. 2008; 266: 164–166. 10.1016/j.jns.2007.09.021 17950753

