# Supplementary figures and images for: Different subregional metabolism patterns in patients with cerebellar ataxia by 18F-fluorodeoxyglucose positron emission tomography
Source: PLoS One. 2017 Mar 20;12(3):e0173275. doi: 10.1371/journal.pone.0173275 (PMC5358749; doi:10.1371/journal.pone.0173275)

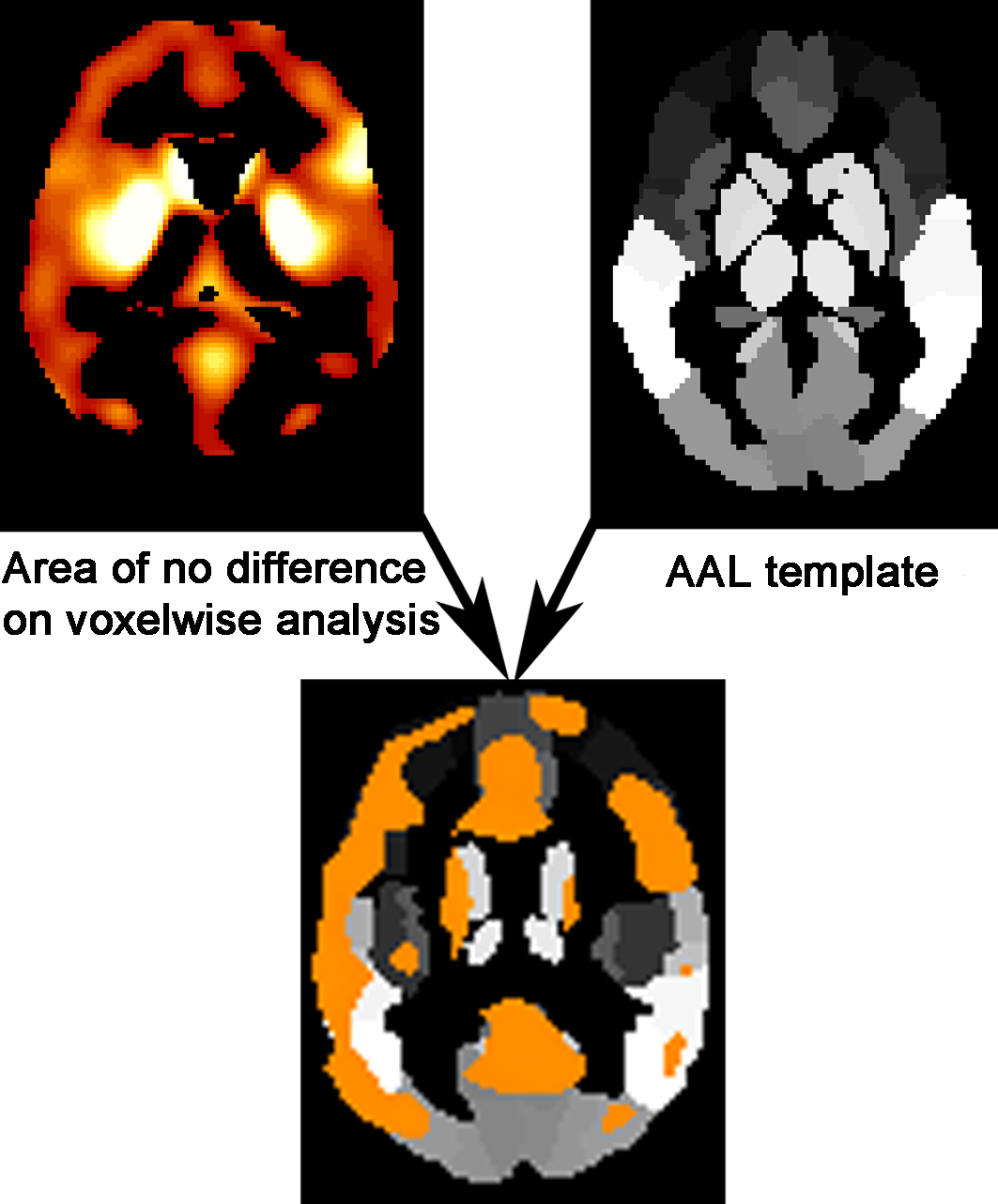

Supplement: S1 Fig — Voxelwise analysis of the disease and control groups (except for crossed cerebellar diaschisis) shows little variance among the groups in parts of the parietal and occipital cortices. (TIF) [file pone.0173275.s001.tif]

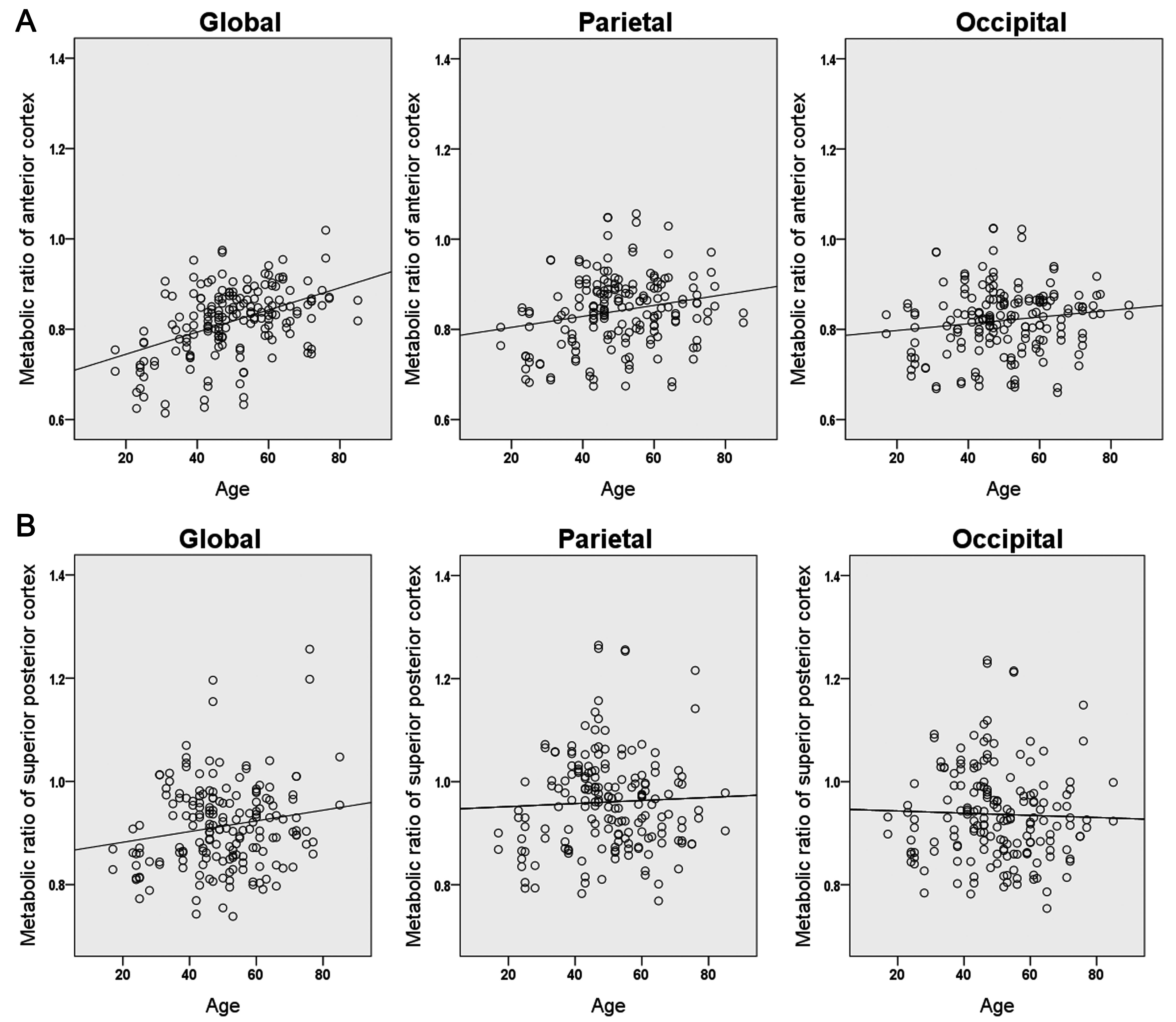

Supplement: S2 Fig — Metabolic ratios of cerebellar subregions with occipital cortex used as reference for count normalization did not change with age (r = 0.141, p = 0.060 for ANT; r = −0.033, p = 0.665 for SUPP) (Right, Figure A and B in S2 File). There was a weak positive correlation in ANT (r = 0.214, p = 0.004) but no correlation in SUPP (r = 0.044, p = 0.556) by count normalization with parietal cortex used as reference (Middle, Figure A and B in S2 File). Moderate and weak positive correlations in ANT and SUPP, respectively, were observed by global count normalization (r = 0.448, p < 0.001 for ANT; r = 0.177, p = 0.018 for SUPP) (Left, Figure A and B in S2 File). (TIF) [file pone.0173275.s002.tif]

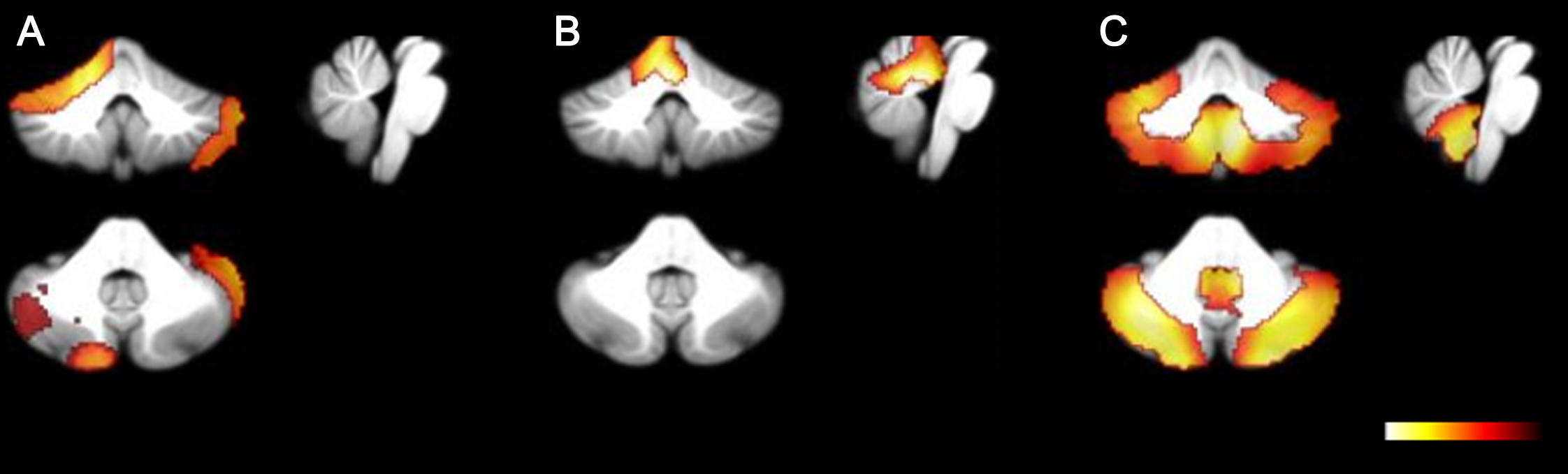

Supplement: S3 Fig — Patients with spinocerebellar ataxia SCA type 2 (Figure A in S3 File) show decreased metabolism in the right anterior and left posterior lobes, compared to those in patients with multiple system atrophy of the cerebellar type (MSA-C). Patients with SCA type 6 (Figure B in S3 File) shows decreased metabolism in the anterior lobe and anterior vermis compared to those in patients with MSA-C. Patients with SCA type 2 (Figure C in S3 File) shows decreased metabolism in the posterior lobe compared to that in patients with SCA type 6. (TIF) [file pone.0173275.s003.tif]
